# MicroRNA Biogenesis, Gene Regulation Mechanisms, and Availability in Foods

**DOI:** 10.3390/ncrna10050052

**Published:** 2024-10-11

**Authors:** Amilton S. de Mello, Bradley S. Ferguson, Erica L. Shebs-Maurine, Francine M. Giotto

**Affiliations:** 1 Department of Agriculture, Veterinary and Rangeland Sciences, University of Nevada, Reno 1664 N. Virginia St. Mail Stop 202, Reno, NV 89557, USA; eshebs@greateromaha.com (E.L.S.-M.); fgiotto@nmsu.edu (F.M.G.); 2 Department of Nutrition, University of Nevada, 1664 N. Virginia St. Mail Stop 202, Reno, NV 89557, USA; bferguson@unr.edu; 3 Department of Animal and Range Sciences, New Mexico State University, Knox Hall 220, MSC 3-I, Las Cruces, NM 88003, USA

**Keywords:** microRNAs, food, nutrigenomics

## Abstract

MicroRNAs (miRNAs) are small, non-coding RNAs that control gene expression by degrading or repressing mRNA translation into proteins. Research recently suggested that food-derived miRNAs are bioavailable and may be absorbed in the gastrointestinal tract (GIT). Since these small RNAs may reach the circulation and organs, possible interactions with host genes will lead to epigenetic effects that alter metabolism. Therefore, from a precision nutrition standpoint, exogenous miRNAs may be essential in modulating health status. This review summarizes the process of miRNA biogenesis, the post-translational mechanisms of gene regulation, and their bioavailability in animal- and plant-derived foods.

## 1. Introduction

MicroRNAs (miRNAs) are highly conserved non-coding RNAs regulating gene expression [1]. These small molecules are initially transcribed from DNA to a primary miRNA (pri-miRNA), cleaved into double-stranded RNA containing from 18 to 23 nucleotides (precursor miRNA, pre-miRNA), and processed into a single strain complex (miRNA plus protein) that binds to messenger RNA (mRNA) [2]. Since miRNAs bind to prime untranslated regions (3′ UTR and 5′ UTR) and silence genes at the post-transcriptional level, they control a broad spectrum of cellular processes, including proliferation, differentiation, development, metabolism, and apoptosis [3,4,5,6].

Recently, the combination of high-throughput sequencing and bioinformatics has allowed for a better understanding of the role of miRNAs in modulating the expression of genes associated with metabolic pathways related to immunity, inflammatory responses, glucose kinetics, obesity, and tumorigenesis [7,8]. Overall, the miRNA expression is regulated by multiple factors, including genetics, dietary patterns, and environmental effects. Since miRNAs are synthesized during DNA transcription, aberrant expressions of these molecules in cells, tissues, and biological fluids indicate that miRNAs can be used as biomarkers for a range of biological indicators, including livestock development and human health [9,10,11,12].

From a nutrigenomics perspective, food-derived miRNAs may also be important in modulating human health since they can regulate gut microbiota, interact with gastrointestinal tract (GIT) cells, and reach target organs after absorption in the stomach or intestines [13,14].

This review highlights details about miRNAs’ synthesis, gene regulation, and their dietary bioavailability in plant and animal foods.

## 2. MicroRNA Biogenesis

MicroRNAs are processed intragenically from introns and exons of nuclear DNA, or intergenically when transcribed from a host gene regulated by their promoters [15,16]. Sections of specific genes (pri-miRNAs) are transcribed by RNA polymerase II or III in long double-stranded clusters denominated pre-miRNAs via the predominant canonical and/or the secondary non-canonical pathway [2,17]. In the canonical pathway, pri-miRNAs are bonded by the protein DiGeorge Syndrome Critical Region 8 (DGCR8) and by Drosha, a class 2 ribonuclease III enzyme [18]. This micro-processing complex composed of DGCR8 and Drosha cleaves the molecule into a smaller double-stranded pre-miRNA. The DGCR8 recognizes areas within the pri-miRNA, including a N6-methyladenylated GGAC and other short sequence motifs, whereas Drosha cleaves the duplex pri-miRNA into a 2 nt 3′ overhang on pre-miRNA [17]. Pre-miRNAs are then exported from the nucleus to the cytoplasm by the protein Exportin 5 and released to have their stem-loop cleaved by the Endoribonuclease III Dicer, which generates an even shorter double-stranded molecule [19]. Both 5p and 3p strands from the pre-miRNA’s 5′ and 3′ ends, respectively, are loaded into the Argonaut protein 2 (AGO 2) [20]. Subsequently, the double strand is unwound, and a single strand is released from the AGO 2. The selection of which strand will remain attached to the AGO 2 depends on the cell’s purpose and environment. However, the strand with lower 5′ stability or 5′ uracil usually becomes the predominant strand that remains loaded into AGO 2 (guide strand). In contrast, the passenger strand (unloaded strand), usually the 3p one, is cleaved by AGO 2 and subsequently degraded by cellular mechanisms [21,22]. MicroRNAs produced via non-canonical pathways are also derived from pre-miRNAs generated by Drosha, DGCR8 [15,16], and Dicer activities. However, the pre-miRNAs are directly exported to the cytoplasm by Exportin 1 without Drosha cleavage [21]. Differently, in this pathway, the 3p strand becomes the guide strand since a 7-methylguanosine (m7-G) cap in the double-strand pre-miRNA usually prevents the 5p strand from being the primary strand loaded into the AGO 2 [22]. Drosha also produces some pre-miRNAs from endogenous short hairpin RNA transcripts, and due to their short length, the AGO 2 completes their maturation in the cytoplasm by binding to both strands and building a single strand after trimming the 3′–5′ sequence of the 5p [23,24].

## 3. Gene Regulation

MicroRNAs regulate gene expression via decapping, deadenylation, and translation repression of targeted regions of the mRNA. Interactions with 5′ UTR are associated with silencing effects and interactions with other areas with induced transcription [25,26,27]. The one strand loaded into the AGO 2 forms an RNA Induced Silencing Complex (RISC) that combines itself into a complementary existent sequence of the mRNA-denominated miRNA response elements (MREs) [26]. Once paired, the AGO 2 endonuclease cleaves regions of the mRNA. Proteins including the GW 182, PAN2-PAN3, CCR-NOT, and DCP2 promote deadenylation and decapping of the mRNA, followed by a 5′–3′ degradation promoted by Exoribonuclease 1 [28,29,30]. Translation is inhibited by preventing the ribosome subunit from binding the mRNA [29]. Those mechanisms avoid protein synthesis, leading to gene silencing. Research also suggested that miRNAs can mediate transcription and gene regulation within the nucleus since AGO 2 may re-enter the nucleus using the Importin-8 and being guided by a GW182 protein (TNRC6A) [24]. The RISC not only regulates the transcription of mRNA in its on-cell, inducing degradation, but also suppresses the transcription of genes regulated by other pathways, including integration with stem-loops of DNA strands and genomic loci [31,32].

## 4. Availability in Foods and Suggested Absorption Pathways

Overall, the ability of an RNA strand to maintain its integrity and withstand degradation in vivo depends on nucleotide sequences and their modifications [31]. Those rules are usually applied to coding and non-coding RNAs and include more than 170 modifications, determined mainly by the action of a writer, binding, and eraser protein [33,34,35]. Dietary RNAs from plants, animal tissues, and fluids such as milk must resist post-harvest practices and digestion to become bioavailable. Post-harvest practices include tissue aging and thermal treatments such as refrigeration and cooking. Once ingested, solid food is mechanically fragmented and chemically processed by GIT enzymes that break carbohydrates, proteins, and fat. The same happens with liquid foods such as milk, which undergo chemical digestion. Thus, the ability of RNA molecules to withstand various environmental stresses and maintain their functionality plays a vital role in their bioavailability and further absorption.

Previous studies have demonstrated that RNA stability may remain constant for several days postmortem, depending on the tissue [36,37,38]. In addition, the higher the amount of G and C in the sequence, the more resistant the strand is to degradation [37]. In contrast, notable degradation of RNAs can be seen over time, which is crucially affected by the strand length and temperature [39,40]. RNAs have many hydrogen bonds, which are very sensitive to temperature. The thermal energy disrupts the electrostatic attractions, holding them together and reducing the bonding interactions. Unlike RNAs, miRNAs seem more stable at high temperatures [8]. Their resilience and ability to be absorbed are associated with a protection effect provided by the AGO2 protein of the RISC complex and the carrier function of vesicles called exosomes.

As previously discussed, miRNAs are functional AGO2 proteins, part of the RISC complex [20]. Those proteins protect miRNAs from target-directed degradation, usually mediated by ZSWIM8 ubiquitin ligase [41,42]. Dietary miRNAs are packaged in exosome and exosome-like vessels in tissues and fluids used as foods. Exosomes are 30–160-nanometer vesicles of a phospholipid bilayer membrane, secreted by cells, carrying DNA, RNAs, cytokines, and proteins [43,44]. Besides offering protection to miRNAs, exosomes also have an affinity for intestinal epithelium cells. They interact with and bind to these cells, delivering their cargo by internalization [43]. The mechanisms of absorption of miRNAs will be further discussed in this review.

As previously discussed, most of the dietary miRNAs are packaged in exosomes. These vesicles protect the strands from the severe conditions of the GIT, including the low-pH environment of the stomach [44]. This protection effect ensures that miRNAs stay stable and bioactive before being absorbed. Overall, miRNAs can be absorbed in the GIT through SID-like transporters, by vesicle-mediated transcytosis, ribonucleoprotein complex-mediated endocytosis, immune cells present in the gut barrier, or diffusion in the space between epithelial cells [45,46]. Plant miRNAs are packaged into exosome-like nanoparticles. Exosome-like nanoparticles also serve as a protective barrier against the degradation of miRNAs but are internalized differently from mammalian exosomes. Although they have not been deeply investigated as mammalian exosomes, research showed that they also increase intestinal epithelial cells’ proliferation [45], directly contributing to intestinal cell longevity.

### 4.1. Dietary miRNAs and Evidence of Circulation Delivery

Although the absorption of miRNAs from food is controversial, previous research has reported the cross-kingdom transfer of dietary plant miRNAs to mammals via oral ingestion. Those miRNAs reached the circulating plasma, culminating in a post-transcriptional regulation of gene expression in humans [46]. This study led scientists to hypothesize that miRNAs from exogenous animal and plant food sources can be absorbed in the GIT and modulate the metabolism of subjects consuming these foods.

#### 4.1.1. Plant Dietary miRNAs

Studies have suggested that plant miRNAs available in fresh produce are resistant to high temperatures and degradation in acidic conditions such as the environment of the GIT [47,48,49,50,51]. Liang et al. [52] demonstrated that dietary miRNAs from watermelon were expressed in its juice after 1 h of preparation. MicroRNAs from watermelon juice, including *ath*-miR 166a, *ath*-miR 390a, *ath*-miR 168a, *ath*-miR 157a, *ath*-miR 162a, and *ath*-miR 172a, were present in the subjects’ plasma for at least 9 h after they consumed the juice. When evaluating healthy male and female subjects who consistently consumed rice-rich diets, Zhang et al. [46] found the expression of about 30 known plant miRNAs in their serum. Among those, the authors observed homologous miRNAs, similar to those detected by Liang et al. *osa*-miR 168a directly affected cholesterol regulation by lowering the expression of the LDLRAP1 gene in the liver. However, although those findings provided evidence of the uptake of plant-derived miRNAs by human epithelial cells, Micó et al. [48] did not detect the expression of exogenous plant miRNAs in the circulation and organs after humans consumed olive oil and beer. In this case, the absence of miRNAs in the plasma was associated with a shallow expression of those molecules in olive oil and beer. Although miRNAs possess resilient properties against processing steps such as high temperatures and lower pHs, it seems that the high-processed nature of those products lowered the concentrations of miRNAs. Snow et al. [50] evaluated the bioavailability and absorption of three plant miRNAs in human subjects. The authors assessed the expression of miR 156a, miR 159a, and miR 169a in the plasma of athletes who consumed diets comprised of cantaloupe, apple, banana, and orange, fruits presenting a substantial expression of those plant miRs. Plasma evaluated 24 h postprandial showed undetected expressions of those three highly conserved plant miRNAs. In this last study, although the food sources had high expression levels of plant miRNAs, we postulate that the expected expression of those miRNAs in the plasma was not attained due to the lower amounts of fruits ingested by the athletes and the time interval between food consumption and postprandial plasma harvest. In the previous studies that demonstrated the absorption of exogenous miRNAs [47,48], subjects received either higher or consistent amounts of plant-derived foods.

#### 4.1.2. Milk Dietary miRNAs

The microRNA profile of milk varies depending on the stage of lactation, which is divided into two phases, producing either colostrum or mature milk. Colostrum has higher concentrations of miRNAs related to growth, development, and the immune system, making breastfeeding an important activity for infants’ development [51]. Wu et al. [53] detected 782 miRNAs in human colostrum, whereas 67 were colostrum-specific miRNAs associated with the oxidative stress system, inflammation, development, and longevity. Those findings suggest that colostrum miRNAs modulate infants’ development and lifespan. Overall, miRNAs in raw milk are primarily contained in exosomes, suggesting that those are protected against degradation [54]. However, commercial milk undergoes homogenization and thermal treatments, most commonly pasteurization or ultra-high-temperature processing (UHT) [55]. Those processing steps can alter the amount of miRNAs present in the milk. While homogenization causes the degradation of miRNAs due to the changes in pressure [56], damaging or disrupting the exosome membrane, temperatures used for pasteurization (85 °C for 15 min) and UHT treatments (135 °C for 15 min) lead to miRNA denaturation. Still, the total read counts of miRNAs in raw, pasteurized, and UHT-treated milk were within 7 log, suggesting that miRNAs, especially the longer ones containing higher GC percentages, are abundant in treated milk [57]. Nonetheless, while their presence in milk is confirmed, the uptake of dietary miRNAs by the GIT in humans remains uncertain. Baier et al. [58] postulated that the bioavailability of miRNAs conferred by exosome protection, their large availability in commercial milk, and the consistent and high consumption of this animal product by many humans could serve as determining factors leading to the transfer of miRNAs to the circulation. The authors reported an increase in miRs 29b and 200c in human plasma 3.4 to 4.2 h postprandial.

Furthermore, milk exosomes increased the expression of RUNX2 in peripheral blood mononuclear cell cultures. However, the evidence suggesting that milk is a potential source of miRNAs that can be absorbed in the GIT and modulate human genes has a considerable implication from a nutrigenomics standpoint. These initial findings led other authors to investigate absorption pathways and how bioinformatics data were interpreted. Conversely, Auerbach et al. [59] found no traces of both miRNAs in human plasma following milk consumption. The miRNAs detected by Baier et al. may be analogous to humans [60], but the authors argued that the miRNAs found in human plasma after milk ingestion are not derived from food sources, and that their presence reported in animal tissues was due to errors of bioinformatics and data interpretation.

#### 4.1.3. Meat Dietary miRNAs

Unlike milk, very little research has been conducted to evaluate the bioavailability of miRNAs in animal-tissue-derived foods. Muscle and fat tissues are usually aged from 7 to 14 days before reaching the consumer, who cooks the tissue before consumption. These treatments involve cold storage and heating, which may affect the tissue’s miRNA content. In fresh beef, the expression of miRNAs in the m. longissimus dorsi varies according to the fat content of the muscle and its location. Studies reporting miRNA expression in unaged beef revealed that miRNAs-145,143, 1246, and 23b-3p are usually higher in intramuscular fat, whereas miRNAs-2325c, 3616, and 2361 are overexpressed in subcutaneous fat [61]. However, Dever et al. [62] reported that human homologous miRNAs are available in unaged cooked beef. Although miRNA expression of miRNAs reduced by between 20% and 50% after cooking, the authors determined that miRNAs in muscle tissue are resilient and resist thermal treatments. The most predominant miRNAs were 10b-5p, muscle-specific miRNA-1, and miRNA-206. As mentioned, meat undergoes an aging process before reaching the final consumer. For a better eating experience, consuming beef 14 days postmortem is recommended since this period allows for better enzymatic tenderization [63]. Therefore, to correctly estimate the dietary availability of miRNAs in beef, it is necessary to simulate the aging process after the animal is butchered and evaluate the miRNA expression of cooked samples. Although novel research still needs to be conducted to assess the resilience of miRNA in aged cooked beef, there is scientific evidence that miRNAs may survive aging. Some miRNAs are overexpressed in cardiac muscle 1 and 4 days postmortem [37] and, therefore, they may be available in commercial beef. Regarding other meats, Shen et al. [64] demonstrated that isolated exosomes extracted from pork survive thermal treatments and when administered to mice, they may be absorbed in the GIT. The authors suggested that miRNAs present in the exosomes can reach the circulation and alter the transcriptome of mice. However, the pork-derived miRNAs showing higher expression in the blood were also homologous to mice, raising similar questions as posed by Snow et al. [50] who argued that milk-derived miRNAs found in the circulation were homologous to miRNAs’ human subjects. In summary, further and detailed research is needed to understand the kinetics of meat-derived miRNAs in the GIT.

## 5. Conclusions

It was proposed that dietary miRNAs act as functional nutrients in modulating the gene expression of humans. Although this hypothesis was tested and accepted when evaluating the kinetics of plant-derived miRNAs, the nutrigenomics impact of exogenous dietary miRNAs from animal foods remains uncertain. Existent data suggest that animal-derived miRNAs are available and protected in food products, even after refrigeration and high-temperature treatments. However, due to their homology with hosts’ miRNAs, their presence in the plasma does not directly suggest their absorption. Elucidating whether dietary miRNAs can regulate the expression of genes modulating diseases and physiological conditions seems to present a new challenge in dietetics, especially animal-derived miRNAs. With advances in DNA and RNA editing in plants and livestock, it will become possible to design foods with an optimal molecular composition that favors health and improves longevity. However, before exploring this new frontier, novel research must be conducted to improve our understanding of how exogenous miRNAs reach the circulation, target organs, and participate in post-translational processes modulating the host gene expression.

## Data Availability

All data generated and analyzed during this study are included in this published article.
